# Putting measurement-based care into action: A mixed methods study of the benefits of integrating routine client feedback in coordinated specialty care programs for early psychosis

**DOI:** 10.21203/rs.3.rs-3918063/v1

**Published:** 2024-02-08

**Authors:** Piper Meyer-Kalos, Grace Owens, Melissa Fisher, Lionel Wininger, Anne Williams-Wengerd, Kimberleigh Breen, Josephine Abate, Ariel Currie, Nathan Olinger, Sophia Vinogradov

**Affiliations:** University of Minnesota Medical School; University of Minnesota Medical School; University of Minnesota Medical School; University of Minnesota Medical School; University of Minnesota Medical School; Minnesota Supercomputing Institute; University of Minnesota Medical School; University of Minnesota Medical School; University of Minnesota Medical School; University of Minnesota Medical School

**Keywords:** Measurement-based care, First episode psychosis, Personalized feedback report, Shared decision-making

## Abstract

**Background:**

Measurement-based care (MBC) is an effective tool in the delivery of evidence-based practices (EBPs). MBC utilizes feedback loops to share information and drive changes throughout a learning healthcare system. Few studies have demonstrated this practice in team-based care for people with early psychosis. This paper describes the development of a personalized feedback report derived from routine assessments that is shared with clients and clinicians as part of a MBC process.

**Methods:**

We used a quasi pre-post comparison design with mixed methods to evaluate the implementation of a personalized feedback report at 5 early psychosis coordinated specialty care programs (CSC). We compared clients enrolled in CSC who did and did not receive a feedback report over the first 6 months of treatment. The sample included 204 clients: 146 who did not receive the feedback report and were enrolled over 2 years, and 58 who received the feedback report. A subset of 67 clients completed measures at both intake and 6-month follow-up, including 42 who received the report and 25 who did not. We compared the two groups with regard to self-reported symptoms, likelihood of completing treatment, and perception of shared decision making. We conducted qualitative interviews with 5 clients and 5 clinicians to identify the benefits and challenges associated with the personalized feedback report.

**Results:**

People who received a personalized feedback report reported significant improvements in shared decision-making and had greater improvements over time in their intent to attend future treatment sessions. They engaged in more sessions for Supported Employment and Education (SEE), case management, and peer support, and fewer medication visits over the first 6 months of treatment. Both groups showed significant improvement in symptoms and functioning. Results from the qualitative analysis indicated that the experience of receiving the reports was valuable and validating for both patients and clinicians.

**Conclusions:**

A personalized feedback report was integrated into standard of care for early psychosis programs. This process may improve shared decision-making, strengthen the likelihood to stay in treatment, and increase engagement in psychosocial interventions. We posit that this process facilitates strengths-focused discussions, enhances intrinsic motivation, and strengthens the therapeutic alliance.

## Background

Measurement-based care (MBC) and evidence-based practices (EBPs) are data-driven approaches to improve outcomes that are a key component of learning healthcare systems (LHS) (1). In MBC, outcome-relevant measures are collected over the course of treatment, shared with clinicians and clients, and acted upon to inform treatment (2–4). It can be conceived of as a real-time “GPS” that uses routine clinical data collection to monitor progress in treatment and outcomes while incorporating client and clinician feedback loops to support change (5). In mental health settings that incorporate MBC, clients experience greater improvements in symptoms, treatment participation, and active decision-making (1, 5, 6). When clinicians participate in MBC, they can more rapidly identify clients who are not making progress in treatment, collaborate with more care providers, and deliver more individualized interventions (5, 7). The integration of MBC with EBPs enhances the shared decision-making process with the aim of providing client-centered treatment informed by research and client progress.

The benefits of MBC extend beyond monitoring improvements in symptoms and identifying treatment non-response. Clients and clinicians who participate in MBC receive more information on a client’s progress in treatment, often leading to greater satisfaction (8). Clinicians note improvements in communication, relationship building, and treatment engagement when MBC is embedded in the treatment protocol (9, 10). As a result, MBC is an important tool to enhance the working alliance between clients and clinicians (11), creating feedback loops that enhance opportunities to collect and share information with the goal of improving treatment. Implementation science suggests that when the feedback loop in MBC is built with training and treatment recommendations that align with current EBPs, teams have opportunities to build competency, increase communication, enhance shared decision making, and identify individualized treatment approaches (12). MBC is thus a powerful therapeutic tool.

Early intervention for psychosis has grown rapidly in the US over the last 15 years with implementation of Coordinated Specialty Care (CSC) teams across the US (13). A recent review of the use of MBC in early intervention programs found that the majority measure a wide variety of outcomes at various intervals in treatment (14), but only a small handful share feedback with clinicians or clients (15–21), and only one program examined the benefits of using an individualized feedback report to provide information about client outcomes for practitioners to use (21). The feedback report was designed for practitioners and not shared with clients in Wong et al., 2006. Practitioners found the feedback process helpful, but they described needing more support to share results of the outcome assessments more confidently and use the results to inform or adapt treatment (21).

Despite these promising early examples, many CSC programs experience challenges in establishing an MBC process, with concerns that (1) clients will decline or be unable to complete the measures; (2) clinicians will have difficulty incorporating assessments and feedback reports into clinical care; and (3) the results will not be specific or helpful to some individuals, or may even be “harmful” (15, 21). Recommendations to improve the process include better integration of MBC assessments into existing clinic workflow, providing information to clinicians about how to use the assessments to inform next steps in treatment, and developing policies to support the implementation of MBC as part of EBPs (12). Unfortunately, a frequently overlooked feature is the guidance needed to translate assessment results into clear and easily comprehensible treatment recommendations informed by EBPs.

In this paper, we describe how we have addressed these issues and developed a client-centered MBC approach within our CSC LHS, as part of our participation in the Early Psychosis Intervention Network (EPINET). EPINET is an NIMH-sponsored initiative to develop a national LHS consisting of “hubs” around the country that deploy MBC within their CSC programs (22). All EPINET hubs use the Core Assessment Battery (CAB) to collect harmonized measures every 6 months on individuals enrolled in their CSC programs, and three hubs have described how they share these data through digital dashboards that CSC team members can access (23–25). Although clients and clinicians participating in EPINET complete CAB assessments every 6 months, strategies to routinely access therapeutically relevant measures and provide helpful individualized feedback to inform treatment have not yet been developed and disseminated. Thus, key components of the MBC mechanisms of action are missing at present.

The purpose of our study was to create, implement, and assess a process that would harness these missing mechanisms of action. Within our Minnesota hub of EPINET (EPI-MINN), we developed a strengths-focused personalized feedback report that presents MBC data to clients and clinicians using a therapeutic focus, encouraging them to use the report to inform personalized treatment. We hypothesized that clients and clinicians could incorporate a personalized feedback report into their standard workflow and that clients who received the report would experience enhanced autonomy and engage in more treatment services.

## Methods

### Overall Study Design

We used a quasi-experimental pre-post comparison group design for our quantitative analyses. 204 participants were enrolled in one of 5 CSC clinics in our EPI-MINN network in the Minneapolis metro area and received CSC services between 2018 and 2023. All clinics provide individual resiliency training, supported employment and education (SEE), family education, case management and medication management according to the NAVIGATE model’s curriculum. Each clinic also includes a psychometrist who acquires LHS measures at intake and on a biannual basis thereafter. During appointments with the psychometrists, clients were informed about the EPI-MINN MBC study and could authorize the use of their measurement-based care assessment data for research. Only those clients who provided authorization are included in our analyses. All participants in the qualitative interviews completed informed consent were given a $30 gift card for their participation. Capacity to consent in clients was assessed using the University of California, San Diego Brief Assessment of Capacity to Consent (UBACC) (26). All procedures were approved by the University of Minnesota Institutional Review Board.

### Setting

Our EPI-MINN hub includes five CSC programs in the state of Minnesota which are part of the EPINET consortium. These programs serve approximately 200 individuals a year. Four of the five clinical sites are located in an urban area; one site is located in a suburban area in northern Minnesota. Each program follows the NAVIGATE model’s curriculum, offering Individual Resiliency Training (IRT), supported employment and education (SEE), family education, case management, and medication management to individuals ages 15–40 who have experienced a first episode of psychosis (27). In addition, each site includes a psychometrist, who obtains the MBC measures, including the EPINET CAB (as standard of care) from clients at program enrollment and every 6 months thereafter until discharge or graduation.

### Participants

Participants were recruited from all 5 EPI-MINN CSC programs. The CSC program criteria includes individuals ages 15 to 40 with a diagnosed psychotic disorder; two of the programs require an onset within the past two years and three accept clients with onset within the past five years. A total of 204 clients were included in the quantitative analyses; 133 had data before the implementation of the personalized feedback report. Data was collected as far back as January 2018 for these 133 clients. 71 clients had data collected after the implementation of the feedback report (December 2021). The data for the quantitative analysis includes all data gathered up until December 2023. Of the 204 clients in the sample, 146 clients did not receive the feedback report and 58 clients did receive the feedback report. Demographic and clinical characteristics are listed in Table 1. A smaller sample of 67 clients completed self-report measures at both intake and 6 months, and clinicians completed ratings on functioning at both intake and 6 months for 106 of the clients. The number of clients in each sample is listed in a supplementary table **[see Additional File 1].**

Qualitative interviews were performed with 5 clients who had reviewed a feedback report with a clinician in the past three months. Five interviews were also performed with clinicians. Interviews were conducted via Zoom and were recorded and transcribed for analysis.

### Procedures

Upon enrollment in one of our CSC programs, clients were approached by a psychometrist to complete self-report and interview-based measures as part of standard clinical practice. The only exception occurred when a clinician identified that the client was experiencing significant clinical instability. Completing the measures was voluntary, and clients were provided an explanation about MBC and how the MBC assessments were used in their treatment and feedback report.

Clients completed assessments in-person and/or remotely using the Mirah measurement-based care system (28), and a digitalized data acquisition platform developed in-house. The full MBC battery took 1–2 hours for clients to complete. After clients completed the assessments, their feedback report was generated and uploaded to secure storage where all clinicians on the team could access it.

### Development of the Personalized Feedback Report

Prior to the development of a more extensive personalized feedback report, the research team piloted use of a brief report with clients at two CSC sites between 2018 and 2019. This initial report consisted of clinical, cognitive, and motivation measures selected by co-authors Fisher and Vinogradov based on their prior research on the importance of these features for client outcomes and their malleability in response to behavioral and cognitive training interventions (29–33). The process was refined between 2020–2021 based on suggestions from clients, family members, clinicians and clinical teams. The goal was to ensure the creation of a report that presented an easy-to-understand set of actionable findings without placing undue time/effort burden on clients and clinicians. Key features of the report are outlined in a supplementary table **[see Additional File 2]** and an example is presented in a supplementary figure **[see Additional File 3].**

The measures included in the final version of the personalized feedback report are a combination of our original symptom/cognition/motivation measures; EPINET CAB measures, and program evaluation measures required by our EPI-MINN sponsors in the Minnesota Department of Human Services. The measures are listed in a supplementary table **[see Additional File 4].** Each measure in the report is presented with three pieces of information: (1) a description of the measures; (2) a visual representation of the client’s results on the measure, such as a bar graph or normal curve; and (3) personalized recommendations based on the client’s scores. The report is 11 pages long, and begins with measures that are more intuitively easy to understand– symptoms and coping strategies– and progresses to measures that clients may be less familiar with (cognition and motivation). Examples of recommendations in the report include sleep hygiene tips, encouragement to exercise or to discuss medications with the prescriber, or specific modules from the NAVIGATE manuals **[see Additional File 3].** The report is generated through our LHS MBC database, using code that has been developed to automate the report for a single time point for an individual client. The personalized feedback report was available for clinicians to use beginning in December of 2021.

The authors (PMK, MF, LW, and SV) developed a protocol and training manual to teach clinicians how to interpret and deliver the personalized feedback reports to clients and families in a patient-centered, strengths-focused manner. Clinicians were explicitly encouraged to view the reports as “conversation starters,” to highlight both absolute and relative strengths, and to discuss deficits and severe symptoms as potential targets for treatment. The Findings Visit training utilized principles of self-determination theory to increase adoption; clinicians gained confidence by any combination of: reading the manual, watching the training video, shadowing visits or having their own shadowed by a clinical psychologist (LW). Continuous support was offered to clinicians in their individual work with clients. Some CSC team members have no formal training in psychological assessment, so additional training sessions were offered, such as role plays and reviews of real clients’ data. Teams at the five programs varied in how they chose to present feedback reports to clients. One team incorporated the report into a treatment planning visit completed with the client and multiple team members shortly after their diagnostic assessment and intake. Other teams chose to have a clinical psychologist present it, either during a visit where the client’s primary clinician was present or during a separate consultation with the psychologist. Additional support was needed and provided to teams that chose to integrate the report into the electronic medical record and bill for psychological assessment services.

### Dependent Variables

The CollaboRATE was used to assess the extent to which health care providers engaged clients in shared decision making about their health issues and treatment (34). Clients rated 3 items on a scale of 1 (no effort was made) to 5 (every effort was made). Engagement in treatment services was measured using: 1) the Intent to Attend scale, which asks clients to rate two questions on a scale of 0 (not at all) to 9 (extremely) how likely they will attend the next appointment and how likely they will complete the program (35), and 2) the number of visits from intake to 6 months in IRT, SEE, medication management, case management, family education, peer support, and family peer support. Symptoms were assessed using the Modified Colorado Symptom Index (36). Clients rated 14 items on a scale of 0 (not at all) to 4 (at least every day). Clinicians provided ratings of functioning using the Mental Illness Research Education and Clinical Center version of the Global Assessment of Functioning (MIRECC GAF) (37). The MIRECC GAF includes 3 subscale ratings for symptoms, occupational and social functioning on a scale of 1 (dangerous) to 100 (fully-functional).

Client and clinician ratings of the feedback report were solicited via a QR code/link to an anonymous survey not connected to any individual report data. Five statements regarding the report’s usefulness were graded on a Likert scale, along with open ended questions for additional comments and suggestions.

### Qualitative Interviews

Qualitative interviews lasted approximately 30 minutes and focused on clinicians’ experiences facilitating the feedback session, or clients’ experiences participating in it. Questions asked of clients and clinicians included what was most “meaningful” and “least helpful” about the sessions as well as what may be “missing” from the feedback sessions.

### Data Analysis

Independent samples t tests (2-tailed) or chi-square tests were used to compare the two groups– those who did not receive the report vs. those who did– on demographic variables, symptom severity, and functioning at program enrollment. Repeated Measures ANOVA (rANOVA) was used to compare the groups in the change from intake to 6 months in the, Shared Decision Making, Intent to Attend Scale, the Modified Colorado Symptom Index and the MIRECC GAF. Mann-Whitney U tests (2-tailed) were used to compare the two groups in duration of untreated psychosis and in engagement in treatment services at 6 months of treatment. All variables were screened, and outlying values less than − 2.5 SD and greater than + 2.5 SD from the mean were Winsorized. All analyses were performed using SPSS Statistics version 28 (IBM Corp). For the anonymous QR code survey, we presented basic descriptive statistics in light of low sample size.

### Qualitative Analyses

Analyses of the individual transcripts were conducted utilizing a multiple case study methodology (38) and was performed by 4 members of the research team. To ensure quality and rigor of qualitative analysis, researchers utilized three primary strategies. First, multiple researchers engaged in independent coding of the data to enhance dependability of the analysis (39). Secondly each researcher engaged in independent line by line coding of all transcripts in response to each interview question. All four researchers then met together via Zoom on three occasions to review their independent coding and identify overlapping patterns across codes. Researchers then collapsed these codes into larger themes both within and across client and provider transcripts. Finally, two researchers identified three overall themes across the ten interviews. It should be noted that while researchers were open to identifying unique themes that differed between participant and provider experiences, the themes that were identified were evident in both sets of interviews.

Researchers engaged in member checking to provide credibility of the analysis. To conduct member checks, the interviewer sent each interviewee a copy of the interview transcript by email to request their review of the transcript. Researchers requested that participants review the transcripts for any missing data or information that could be misunderstood by researchers. Finally, researchers utilized triangulation of the qualitative data with the survey data to support the confirmability of the analysis.

## Results

This study took place during the early stages of development of LHS processes within our hub. As a result, the initial data sets contain multiple instances of missing data. Thus, our inferential statistical analyses were carried out on subsamples of the larger data set presented in a supplementary table **[see Additional File 1].**

### Quantitative Results

#### Demographic and clinical characteristics

In the total sample, the groups did not differ in age at enrollment, education, gender, race, diagnosis, number of hospitalizations, or symptom severity. Measures that were only completed by a subset of clients are denoted at the end of Table 1 along with sample sizes. Group differences in duration of untreated psychosis were at trend level significance, with the Yes-Report group showing lower duration of untreated psychosis (DUP) compared to the No-Report group.

#### Self-Report Measures

There were 42 individuals who received the report and 25 individuals who did not receive the report who completed the following self-report measures at both intake and 6 months: Shared Decision Making, Intent to Attend Treatment, and the Colorado Symptom Index. Group differences in demographic and clinical characteristics in this subsample of clients were all non-significant.

#### Shared Decision Making

There was a significant main effect of time (p = .013) in ratings of how much effort was made to listen to the things that matter most about health issues. Both groups felt more effort was made after 6 months of treatment compared to intake (Table 2). Post hoc analyses revealed this was driven by a significant increase in the ratings completed by the Yes-Report group (t(41)=−3.71, p < .001), while the change in the No-Report group was non-significant. There was a group by time interaction at trend level significance (p = .087) in ratings of how much effort was made to include what matters most in choosing what to do next. Post hoc analyses showed that this was due to a significant increase in the Yes-Report group (t(41)=−2.80, p = .008), and no significant change in the No-Report group.

#### Intent to Attend Treatment

There was a group by time interaction at trend level significance (p = .090) in the change in ratings from intake to 6 months of how likely clients would attend their next appointment. The Yes-Report group rated it more likely that they would attend their next appointment after 6 months of treatment compared to intake, and the No-Report group rated it less likely that they would attend their next appointment after 6 months of treatment compared to intake (Table 2). Each groups’ ratings were high (in the “markedly” range) at intake and after 6 months of treatment. There was a significant main effect of time in the change in ratings from intake to 6 months of how likely clients would complete the program (p = .024). Both groups rated they were more likely to complete the program after 6 months of treatment compared to intake. Post hoc analyses revealed that the increase in the Yes-Report group was statistically significant (t(41)=−2.88, p = .006), while the increase in the No-Report group was non-significant.

#### Modified Colorado Symptom Index

There was a significant main effect of time in the change in symptom frequency from intake to 6 months (p < .001). Both groups rated they were experiencing symptoms less frequently after 6 months of treatment compared to intake (Table 2). Post hoc analyses revealed this was driven by a significant decrease in the ratings completed by both the Yes-Report group (t(41) = 1.178, p = .003) and the No-Report group (t(24) = 3.313, p = .003).

#### Treatment Visits

The number of visits was collected for 58 clients who received a feedback report and 146 clients who did not receive a feedback report (N = 204 total). The groups differed significantly in the number of SEE, medication management, peer support, and case management visits. Individuals who received the report attended significantly more SEE, peer support, and case management visits and significantly less medication management visits (Table 3).

#### Functioning: MIRECC GAF

The MIRECC GAF was completed by clinicians for 33 clients who received the feedback report and 73 clients who did not receive the feedback report (N = 106 total). There was a significant main effect of time in the change in occupational, social, and symptomatic functioning from intake to 6 months (p < .001).

Both groups had greater functioning in all domains after 6 months of treatment compared to intake (Table 4). Post hoc analyses revealed that both groups showed statistically significant improvement in occupational, social, and symptomatic functioning: Yes-Report group (t(32)=−5.16 to −3.86, p < .001); No-report group (t(72) = −5.65 to −4.06, p < .001).

#### Feedback Report Survey

Nine clients and thirteen team members filled out the anonymous survey regarding feedback reports. Most clients and team members who responded agreed that the feedback report was easy to read, useful, and that the recommendations were helpful. Team members also strongly endorsed the likelihood of using the report in future appointments. Clients were more neutral about the likelihood of sharing the report or recommendations with family members (Table 5).

#### Qualitative Results

##### Theme 1: Feedback reports and sessions were valuable.

One particular theme that was common across almost all participant accounts was the overall value of the report and feedback session for both clients and clinicians. There appeared to be three overall dimensions of this theme. First, both clients and clinicians articulated the value of the visual and concrete information delivered in the report. While clients reported valuing the “visuals” and “graphics” provided in the feedback report, clinicians endorsed the value of having “concrete” or “objective” information to connect clients to treatment and foster engagement and motivation. Further, clinicians highlighted the value of having “neutral” data to discuss with their clients; one stated, “You know, we’re having lots of conversations about treatment and about. .. symptoms and functioning but it [the feedback report] provides something that is a bit concrete or tangible. .. this is a tool that doesn’t really exist in another way[.. .].”

Second, reviewing personalized feedback reports may contribute to building a growth mindset. Both clients and clinicians pointed to the value of the information gained from the reports over time. One client participant stated, “. .. just being able to see the growth that I’ve had from the first time I was assessed really validated the skills I’ve been learning in therapy and the benefits of getting my housing more situated. I was able to graphically see my improvements.” Similarly another client participant stated, “I think what was helpful most is that I’ve taken this feedback study multiple times so I’ve been able to see how things have changed. So I think that was one of the biggest benefits.” Clinicians likewise reported value from seeing changes over time including improvements and being able to “celebrate” with their clients as well as provide confirmation of symptoms or struggles they might be experiencing. This theme suggests that the personalized feedback reports may help clients see improvement over time leading to more opportunities to build motivation.

Third, clinicians and clients described that engaging in the feedback sessions helped in building rapport and improved communication with them. One client stated, “It just felt like they, you know, they truly cared about me and they care [about] my well-being and me getting better. .. it felt really personal.” Some clinicians mentioned how the feedback session helped them to have more “empathy” for their clients as well as a way to “gain insight” on their clients’ experiences. Another clinician stated that the feedback sessions helped by “. .. being able to tailor your approach and kind of build a therapeutic alliance and get buy-in from the client[.. .].”

#### Theme 2: Feedback sessions were validating

Engaging in the feedback sessions was validating for both clients and clinicians. In particular, clients reported feeling validated in the struggles with cognitive performance or coping as well as in the improvements that they were experiencing over time. One client stated, “I think the most meaningful one was the part that had to do with my cognition because I felt that my memory had been getting a lot worse. .. I noticed that [the] score went significantly down and I’m glad that I saw that because I kind of had a suspicion that that was the case.” Another client stated about the feedback session, “So to have a doctor be like, look at the growth that we’re seeing here, here, and here. It just validated me quite a bit.” Creating more opportunities to validate and show improvements could help contribute to a growth mindset.

Clinicians described the reports as being validating of their work. One clinician stated that it helped in “feeling more confident” on where they can encourage clients in their treatment goals and how to “use the IRT program to be specifically individualized and tailored to their [the client’s] current needs.” The increase in confidence could help clinicians increase their competence to actively use the data from the personalized feedback report in treatment. Clinicians also described noticing how the feedback sessions were “validating” for clients related to their progress over time and how the sessions themselves offered “space to validate the client’s experiences.”

#### Theme 3: Personalizing feedback reports may help both clinicians and clients

Clients and clinicians also spoke of the potential value in tailoring the reports and the feedback sessions for specific clients’ needs. The suggestion of personalizing or tailoring feedback reports was articulated by a few clinicians who described the tension of having too much versus too little information communicated during the feedback session. One clinician stated,

The more information that you have, the more you can talk about. .. which can be really helpful. At times it can also be overwhelming as well, knowing how much information to share at a time and how...scheduling enough time to talk about that information in a session, I think are skills I’m still figuring out.” Another clinician described the value of including other perspectives in the feedback session in particular circumstances when the feedback report is conflicting with clinician observations. This clinician stated, “There seems to be just such legitimacy to everything that’s on there and my concern is that somebody looks at that measure of psychiatric symptoms and they say there are no elevations and it sort of–especially for folks with low insight–it sort of confirms this idea of like, ‘See, I’m totally fine.’

More specifically, client participants in this study described having too much or being “overwhelmed” with information and at other times wanting additional information in the reports. Specifically, multiple clients spoke about receiving information about substance use and how they did not find this valuable because it didn’t apply to their particular experiences. Another participant described the value in balancing information in the report by describing the feedback on coping mechanisms in saying, “I think for me personally it wasn’t helpful, just cause. .. I have some self-awareness, but I think it could be helpful for other people I think–it kind of depends on each person.” Clinicians likewise mentioned having additional examples about things like “cognitive skills” or providing examples of questions which reflect why a particular client’s score was elevated or low.

## Discussion

The strengths-focused personalized feedback report piloted within our EPI-MINN network was designed to provide actionable, clinically relevant information to clinicians and clients in an easy-to-understand format. It was used to provide personalized measures that help to identify treatment target areas and useful treatment strategies, similar to offering different routes to a destination when you plug in an address on your GPS.

Results suggest that clients who received a personalized feedback report showed several interesting outcomes compared to those who did not: shared decision-making, increased intent to attend and engage in treatment, symptoms, and functioning.

### Shared Decision-Making

Clients who received a feedback report showed a greater trend towards feeling that their struggles were being heard compared to the no feedback group. Results from the qualitative interviews suggested clients felt validated by the reports which aligns with improvements to the shared decision making process. Research suggests that guidance on how to use MBC to increase shared decision making varies widely (40). Our results suggest that a personalized feedback report with individualized treatment recommendations may be one approach to increase shared decision making. Outcome measures collected as part of MBC in this project demonstrated the benefits of developing a feedback report that clinicians can use with minimal training and encouragement. The EPINET project has provided opportunities to standardize data collection in more than 100 clinics in the US (41) but integrating data into the clinical decision-making process is challenging. Qualitative feedback suggests that the information was accessible to clinicians and clients and they were able to have meaningful conversations to modify or adapt treatment based on the MBC results. Using a format that incorporates simple data visualizations and provides simple explanations along with concrete recommendations to action steps and EBP treatment strategies may help overcome challenges associated with using MBC data in a shared decision making process (21). The feedback report has shown promising results that could inform clinical decision making and be used as a tool to increase engagement in CSC programs.

### Increased Intent to Attend Treatment

Clients who received the feedback report showed a greater trend towards improvements from intake to 6 months in their self-reported intent to attend their next appointment. Both the Yes-Report and No-Report groups showed an increase in their intent to complete the treatment program, however post hoc analyses indicated this increase was only significant in clients who received a feedback report. One possible interpretation of these findings could be that providing feedback about symptoms, motivation, and cognition at intake to clients with reservations about treatment may validate their illness experience and as a result lead to greater intent to attend and participate in practical, real-world services such as SEE, peer support, and case management. Improving engagement is a common concern facing most CSC programs (42, 43). We found some initial evidence in support of the value of validation from the qualitative interviews where both clients and clinicians reported feeling validated after reviewing the findings.

### Number of Visits Attended

Clients receiving a feedback report attended more SEE, case management, and peer support visits than those who did not receive a report. However, the feedback report group attended fewer medication management visits than the no report group. A common struggle in young people in early intervention treatment programs is disengagement from treatment services, with approximately one-third of individuals disengaging (42). When we support a person’s autonomy, competence, and relatedness, we help them build motivation for change and action according to self-determination theory (44, 45). These findings suggest that when clients receive a personalized feedback report that includes targeted messages about strengths and areas for improvement, they may be empowered and motivated to connect with peers and seek help with getting their needs met at work and school. Increasing the number of visits with these team members could have provided more opportunities to identify work and school goals or apply for work or school programs or problem-solve common challenges. MBC and feedback reports have had mixed findings on improving client outcomes, and this is a very preliminary finding that needs to be confirmed in future studies (46, 47).

Despite the increases in treatment engagement, clients who received a feedback report attended fewer medication visits. This was surprising, but it is possible that the individuals who received a feedback report could have been experiencing fewer symptoms or more positive results from their medications and thus required less frequent medication visits. Research has indicated that clients with first episode psychosis who have a lower severity of illness at baseline are twice as likely to disengage (48). However, there were no demographic or clinical differences from the two groups and their use of medication was similar.

### Symptoms and Functioning

Clients in both groups, who did and did not have a feedback report generated, had similar decreases in symptoms and improvements in functioning, suggesting there was no significant additional benefit to either when utilizing a feedback report. All clients received NAVIGATE interventions, which have been found to improve symptoms and treatment engagement (49). MBC programs do not always lead to significant improvements in symptoms, as has been shown with mixed results in prior studies, and there is less information about how MBC could help improve client functioning over time (46, 47).

Findings from the qualitative interviews and satisfaction survey support the results suggesting that the feedback report is a helpful engagement tool. Results from the current study expand on a previous study where there were mixed reviews about the use of MBC (15). In the current study, clients reported that the personalized feedback sessions were valuable, and the results presented in the reports validated their experiences and created opportunities to see growth. Clinicians noted that utilizing the personalized feedback report helped them build a stronger rapport with their clients. Clinicians in behavioral health and youth mental health settings have reported that MBC could help improve communication, engagement, and alliance (11, 47, 50). Taken together these findings suggest that one possible pathway to increase engagement and motivation is to include opportunities in treatment to validate and discuss the experience of illness. MBC designed as a feedback loop could create more of those instances to share information, and a personalized feedback report could be a tool to connect and discuss current experiences and next steps in treatment.

The practice of MBC can have greater influences beyond treatment retention and reductions in symptoms. Providing the full range of MBC as an EBP has been suggested to include improvements in communication, engagement, alliance, and facilitating a discussion from multiple perspectives (47). We designed the personalized feedback report to help clients make informed treatment decisions. The personalized feedback report presents information that validates an individual experience and presents EBP treatment-informed recommendations to offer choice, educate about best treatment practices, and encourage clients to take a step towards their goal with the support of the CSC team and family members.

### Limitations and Future Directions

There were several limitations to the current study that limit the interpretability of our findings, while also providing direction for future MBC research. First, this naturalistic study did not randomly assign clients to receive a personalized feedback report. Most of the clients who did not receive a report were enrolled at the earlier stages of our LHS development, before the feedback report was implemented. Due to this, there is a possibility that this was a biased sample though there were no significant demographic or clinical differences between the groups. Because the data was collected in a naturalistic setting, there were also differences in the samples that completed the self-report and were rated on functioning. It is also important to note that all of our CSC clinics came from the same Midwestern state, and implementation of the feedback report in other clinics may produce different results. Secondly, we did not measure how each individual clinician used the report, though our own experience and the report generated by Wong et al. suggest that clinicians may need support to discuss MBC findings with clients, and support needed may vary across clinicians and sites (21). Another possible limitation was that data collection began during the pandemic for the No-Report group, when everything had to be collected remotely. There may have been some differences in data collected for the 2 groups since the Yes-Report group began data collection later in the pandemic. Finally, there were low rates of engagement in both groups for the peer support services. These low rates may have been due to staffing challenges across CSC teams or challenges during the pandemic, but may be different if CSC teams remained fully staffed and operated without restrictions for in person meetings.

Future studies should consider how to respond to concerns raised by clients and clinicians. Clinicians suggested the need for increased ability to tailor the reports to the client’s needs. Clients reported a concern that they felt overwhelmed by the information at times. Simplifying the personalized feedback report to adjust the number of findings included in the report could be helpful. For example, if a client does not have a problem with substances or sleep problems, the visualizations could be removed to shorten and focus the report. The feedback report was a clear marker for discussing change, and next steps for bringing information to clinicians and clients will be to present more than one time point in a feedback report. However, keeping the focus on tailoring the personalized feedback report and targeting key outcomes so as not to overwhelm clients or clinicians could improve its utility. Some research has suggested that building motivation for a client’s change over time can lead to increased motivation through autonomy support and linking individual goals to findings in a personalized report (51).

## Conclusions

This pilot study provides initial evidence that measurement-based care coupled with personalized feedback reports can improve early psychosis treatment. Clients felt the report validated their experiences, and participated in more SEE, case management, and peer support sessions. Clinicians gained insight into individual illness experiences and felt more confident in their treatment approach. We generated an automated feedback report, provided training to clinicians, and supported the integration of the reports into treatment. We are hopeful that continued efforts to create these feedback processes will continue to expand opportunities for increases in motivation, greater treatment engagement and progress towards personalized recovery goals. More work is needed to further individualize the reports, especially to patients with low motivation and low insight.

## Figures and Tables

**Table 1:** Demographic and clinical characteristics of personalized feedback report groups (n = 204)

	Yes-Report (N = 58) Mean (SD)	No-Report (N = 146) Mean (SD)	T-test or Chi-Square (p)

Age at entry (years)	22.34 (6.24)	22.04 (4.93)	5.279 (.741)

Education (years)	12.60 (2.38)	12.40 (1.97)	2.187 (.541)

Gender^a^	31 (53.4%)	98 (67.1%)	3.362 (.186)
Male N (%)	25 (43.1%)	44 (30.1%)	
Female N (%)	2 (3.4%)	4 (2.7%)	
Non-Binary or Other N (%)			

Racial background n (%)^a^	17 (29.3%)	23 (15.8%)	9.34 (.155)
Black/African American N (%)	32 (55.2%)	87 (59.6%)	
White N (%)	2 (3.4%)	10 (6.8%)	
Asian N (%)	0 (0.0%)	3 (2.1 %)	
American Indian/Alaskan Native N (%)	7 (12.1%)	15 (10.3%)	
More than 1 race N (%)	0 (0.0%)	2 (1.4%)	
Prefer not to say N (%)	0 (0.0%)	6 (4.1 %)	
Unsure/donť know N (%)			

Diagnosis^a^	6 (10.3%)	20 (13.7%)	4.93 (.553)
Schizophrenia N (%)	9 (15.5%)	33 (22.6%)	
Schizoaffective N (%)	4 (6.9%)	11 (7.5%)	
Schizophreniform N (%)	36 (62.1%)	67 (45.9%)	
Psychosis NOS N (%)	1 (1.7%)	5 (3.4%)	
Major Depression w/ Psychotic	2 (3.4%)	9 (6.2%)	
Features N (%)	0 (0.0%)	1 (0.7%)	
Bipolar Disorder w/ Psychotic			
Features N (%)			
Substance Induced Psychotic			
Disorder N (%)			

Duration of Untreated Psychosis (months)^b^ Median (range)	6 months (130)	9 months (203)	3537.5 (.067)

Hospitalized in the last 6 months^a^	30 (55.6%)	81 (61.4%)	.537 (.464)
Yes N (%)	24 (44.4%)	51 (38.6%)	
No N (%)			
[Yes-Report total N = 54; No-Report total N = 132]^c^			

Modified Colorado Symptom Index mean item score	1.77 (.92)	2.13 (1.39)	.607 (.124)
[Yes-Report total N = 53; No-Report total N = 48]^c^			

MIRECC GAF Occupational Functioning Total	51.60 (26.78)	47.16 (24.71)	1.077 (.283)
[Yes-Report total N = 57; No-Report total N = 114]^c^			

MIRECC GAF Social Functioning Total	64.86 (19.35)	60.72 (19.02)	1.334 (.184)
[Yes-Report total N = 57; No-Report total N = 114]^c^			

MIRECC GAF Symptomatic Functioning	51.95 (19.12)	48.96 (14.85)	1.035 (.303)
[Yes-Report total N = 57; No-Report total N = 114]^c^			

a Chi-Square test.

b Independent-Samples Mann-Whitney U test

c Data were available on a subset of participants.

**Table 2:** Personalized feedback report group comparison of self-report measures.

**Shared Decision Making**	**Yes-Report (N = 42)** **Mean (SD)**	**No-Report (N = 25)** **Mean (SD)**	**Main effect of time**	**Group × time interaction**
How much effort was made to help you understand your health issues? Intake 6 Months	3.93 (0.97) 4.10 (0.82)	3.68 (1.07) 3.84 (1.18)	F(1,65) = 1.074, p = .304	F(1,65) = <.001, p = .983
How much effort was made to listen to the things that matter most to you about your health issues? Intake 6 Months	3.57 (1.25) 4.21 (0.68)	3.56 (1.12) 3.68 (1.25)	F(1, 65) = 6.566, p = **.013**	F(1, 65) = 3.085, p = .084
How much effort was made to include what matters most to you in choosing what to do next? Intake 6 Months	3.40 (1.17) 3.93 (1.02)	3.72 (0.94) 3.68 (1.18)	F(1, 65) = 2.219, p = .141	F(1, 65) = 3.014, p = .087
**Intent to attend**	**Yes-Report Mean (SD)**	**No-Report Mean (SD)**	**Main effect of time**	**Group × time interaction**
How likely is it that you will attend the next appointment? Intake 6 months	6.31 (2.30) 6.67 (2.65)	7.00 (2.00) 6.24 (2.33)	F(1,65) = .384, p = .537	F(1,65) = 2.955, p = .090
How likely is it that you will complete the program? Intake 6 months	5.36 (2.09) 6.55 (2.36)	6.20 (1.89) 6.56 (2.10)	F(1,65) = 5.321, **p = .024**	F(1,65) = 1.527, p = .221
**Modified Colorado Symptom Index**	**Yes-Report** **Mean (SD)**	**No-Report** **Mean (SD)**	**Main effect of time**	**Group × time interaction**
Modified Colorado Symptom Index Mean Item Score Intake 6MO	1.73 (0.93) 1.31 (1.02)	2.05 (1.04) 1.50 (1.00)	F(1, 65) = 20.678, **p = <.001**	F(1, 65) = .408, p = .525

**Table 3:** CSC treatment engagement comparison from intake to 6 months

Treatment Visits	Yes-Report (N = 58) Median (range)	No-Report (N = 146) Median (range)	Mann Whitney U (p)
Individual Resiliency Training (IRT)	14.0 (26)	13.0 (41)	4727.00 (.194)
Supported Employment and Education (SEE)	9.0 (27)	4.0 (23)	5119.00 **(.019)**
Medication Management	3.5 (12)	4.5 (22)	3482.50 **(.047)**
Family Education	3.0 (24)	1.0 (29)	4525.50 (.422)
Family Peer Support	0.0 (3)	0.0 (5)	4579.00 (.218)
Peer Support	0.0 (24)	0.0 (20)	5335.00 **(<.001)**
Case Management	1.0 (15)	0.0 (19)	5394.50 **(<.001)**

**Table 4:** Comparison of functioning between personalized feedback report groups

MIRECC GAF	Yes-Report (N = 33) Mean (SD)	No-Report (N = 73) Mean (SD)	Main effect of time	Group × time interaction
Occupational Functioning Intake 6 months	52.06 (28.28) 75.58 (20.62)	47.67 (25.20) 62.95 (27.72)	F(1,104) = 35.707, **p = <.001**	F(1,104) = 1.612, p = .207
Social Functioning Intake 6 Months	65.45 (19.54) 78.33 (15.39)	59.21 (17.87) 73.01 (16.95)	F(1,104) = 37.385, **p = <.001**	F(1,104) = .045, p = .832
Symptomatic Functioning Intake 6 Months	50.91 (19.98) 66.88 (15.07)	50.25 (14.77) 63.03 (16.86)	F(1,104) = 52.775, **p = <.001**	F(1,104) = .649, p = .422

**Table 5:** Anonymous Feedback Report Survey Results

Survey Item (grade on Likert scale)	Clients agree or strongly agree (n = 9)	Team Members agree or strongly agree (n = 13)
**Overall, I found the feedback session to be useful.**	100% (9/9)	85% (11/13)
**The information and recommendations in the report were easy to understand.**	88% (8/9)	100% (13/13)
**The feedback regarding symptoms was accurate.**	100% (9/9)	92% (12/13)
**The recommendations provided were helpful.**	100% (9/9)	92% (12/13)
**For clients: I will share this information with a family member, friend, or other supportive person in my life.**	33% (3/9)	N/A
**For team members: I will use this information with the patient in future sessions.**	N/A	100% (13/13)

## Data Availability

This data has been shared with the NIMH Data Archive through the EPINET National Data Coordinating Center, and while the dataset is not publicly available at the time of publication, access to the data can be coordinated by contacting the corresponding author.

## Abbreviations

CAB: Core assessment battery
CSC: Coordinated specialty care
DUP: Duration of untreated psychosis
EBP: Evidence-based practice
EPINET: Early Psychosis Intervention Network
EPI-MINN: Early Psychosis Intervention Network - Minnesota hub
IRT: Individual Resiliency Training
FEP: First-episode psychosis
LHC: Learning health care
MBC: Measurement-based care
MIRECC GAF: Mental Illness Research Education and Clinical Center version of the Global Assessment of Functioning
No-Report: People who did not have a feedback report generated
NIMH: National Institute on Mental Health
RAISE: Recovery After Initial Schizophrenia Episode
SEE: Supported Employment and Education
UBACC: University of California, San Diego Brief Assessment of Capacity to Consent
Yes-Report: People who had a feedback report generated
